# The interplay between social and food environments on UK adolescents’ food choices: implications for policy

**DOI:** 10.1093/heapro/daad097

**Published:** 2023-08-30

**Authors:** Sarah Shaw, Sarah Muir, Sofia Strömmer, Sarah Crozier, Cyrus Cooper, Dianna Smith, Mary Barker, Christina Vogel

**Affiliations:** MRC Lifecourse Epidemiology Centre, University of Southampton, Southampton SO16 6YD, UK; NIHR Southampton Biomedical Research Centre, University of Southampton and University Hospital Southampton NHS Foundation Trust, Southampton SO16 6YD, UK; MRC Lifecourse Epidemiology Centre, University of Southampton, Southampton SO16 6YD, UK; MRC Lifecourse Epidemiology Centre, University of Southampton, Southampton SO16 6YD, UK; NIHR Southampton Biomedical Research Centre, University of Southampton and University Hospital Southampton NHS Foundation Trust, Southampton SO16 6YD, UK; MRC Lifecourse Epidemiology Centre, University of Southampton, Southampton SO16 6YD, UK; NIHR Applied Research Collaboration Wessex, Southampton Science Park, Innovation Centre, 2 Venture Road, Chilworth, Southampton SO16 7NP, UK; MRC Lifecourse Epidemiology Centre, University of Southampton, Southampton SO16 6YD, UK; NIHR Southampton Biomedical Research Centre, University of Southampton and University Hospital Southampton NHS Foundation Trust, Southampton SO16 6YD, UK; Geography and Environmental Science, University of Southampton, Southampton SO17 1BJ, UK; MRC Lifecourse Epidemiology Centre, University of Southampton, Southampton SO16 6YD, UK; NIHR Southampton Biomedical Research Centre, University of Southampton and University Hospital Southampton NHS Foundation Trust, Southampton SO16 6YD, UK; School of Health Sciences, Faculty of Environmental and Life Sciences, University of Southampton, Southampton SO17 1BJ, UK; MRC Lifecourse Epidemiology Centre, University of Southampton, Southampton SO16 6YD, UK; NIHR Southampton Biomedical Research Centre, University of Southampton and University Hospital Southampton NHS Foundation Trust, Southampton SO16 6YD, UK; NIHR Applied Research Collaboration Wessex, Southampton Science Park, Innovation Centre, 2 Venture Road, Chilworth, Southampton SO16 7NP, UK; Centre for Food Policy, City, University of London, London EC1V 0HB, UK

**Keywords:** adolescents, food environments, social environments, food choice, qualitative research

## Abstract

Factors from social and food environments can influence the food choices of adolescents in ways not experienced during childhood. Evidence suggests these two environments influence adolescents’ food choices independently, but there is limited knowledge of how the interplay between these environments influence adolescents’ diets. An enhanced understanding of this interplay surrounding adolescent food choice could aid the development of more nuanced interventions and policies. This qualitative study involved 13 online focus groups with adolescents (*n* = 45) aged 11–18 years, attending secondary school or college in England, UK. Data were analysed using thematic analysis. Social experiences which accompanied eating were perceived as more important than the food itself, and fast-food outlets were described as uniquely suited to facilitating these interactions. Young people wanted to spend their money on foods they considered worthwhile, but this did not always relate to the most affordable foods. Adolescents wanted to put little effort into making food decisions and appreciated factors that helped them make quick decisions such as prominent placement and eye-catching promotions on foods they wanted to buy. Chain food outlets were valued as they offered familiar and frequently advertised foods, which minimized the effort needed for food decisions. Adolescents’ sense of autonomy underpinned all themes. Participants described having limited opportunities to make their own food choices and they did not want to waste these buying unappealing ‘healthy’ foods. Interventions and government policies should align with adolescents’ experiences and values relating to food choice to ensure that they are effective with this important age group.

**Contribution to Health Promotion**

- Social environments and food environments are intricately intertwined in driving adolescents’ independent food purchasing practices and facilitate the development of autonomy during this phase of the lifecourse.
- Large chain food outlets, particularly fast-food outlets, offer the right environments for adolescents to make food purchases that align with social needs and affordability.
- Food purchasing behaviours and autonomy should be developed in environments supportive of adolescent health and well-being.
- Future government policies need to incorporate the lived experiences of adolescents to ensure policies are beneficial to young people and unintended effects are avoided.

## INTRODUCTION

The food environment, the point at which consumers intersect with the food system and make decisions about obtaining and consuming food, has been shown to play an influential role in the food choices of all age groups (Downs *et al.*, 2020; Neufeld *et al.*, 2022). However, these environments, especially in high-income countries, have often been described as obesogenic because of the prominence of energy-dense, nutrient poor food (Roberto *et al.*, 2015). Increasing recognition of the important role played by the food environment in determining dietary choices (Caspi *et al.*, 2012; Black *et al.*, 2014; Williams *et al.*, 2014; Shaw *et al.*, 2020; Vogel *et al.*, 2021) has led to the introduction of public health policies that directly alter and improve the healthfulness of the food environment.

In 2010, the World Health Organisation issued recommendations urging governments to develop strong policies to protect children and young people from the marketing of unhealthy foods (World Health Organization, 2012). As part of its Childhood Obesity Strategy (HM Government, 2016, 2018), the UK Government were among the first to introduce a range of policies targeting food environments at national and local government levels. In 2022, the UK national government introduced a policy which requires the calorie labelling of all items on menus and displays in the out-of-home sector, including pre-packaged items such as soft drinks and snacks (Department of Health and Social Care, 2021a). Legislation was also implemented in 2022 to restrict the prominent placement (e.g. at checkouts, store entrances and aisle ends), of foods high in fat, sugar and salt (HFSS) in medium and large food retailers (Department of Health and Social Care, 2021b). Additional legislation to ban volume-based promotions (e.g. buy-one get one-free offers) is set to be introduced in the 2025. Regulations to limit paid-for advertising of HFSS foods on TV and online spaces before the 9 pm watershed are also due to be introduced in January 2025 (Department for Digital, Culture, Media and Sport and Department of Health and Social Care, 2021). Other countries such as Canada, USA and Australia have highlighted the need for policies to address unhealthy food environments and are exploring options for action in these areas (Biden-Harris Administration: The White House, 2022; Commonwealth of Australia, 2022; House of Commons of Canada, 2022). Effective evaluation of these policies will be imperative to understand their impact on the purchasing and dietary behaviours of the population, as such policies may have different impacts depending on age and socio-economic status (SES).

Adolescence is a unique transitional period where individuals gain autonomy over many health-related behaviours including food choices (Bassett *et al.*, 2008). This period of life is critical for health as behaviours established during this stage have been shown to track into adulthood (Craigie *et al.*, 2011). Intervening to improve health-related behaviours during adolescence may have a potential triple benefit: to the adolescent now, in the future, and to any future offspring (Patton *et al.*, 2016). As they experience increased levels of independence, adolescents may, for the first time, begin to make more food choices and purchases independent of adult supervision, using retail environments such as supermarkets, convenience stores, fast-food outlets and restaurants. These environments may therefore play an influential role on the healthfulness of their overall diet.

Physiological changes which occur during adolescence, such as brain remodelling and hormonal changes, are associated with heightened sensitivity to social evaluation and influence (Galván, 2013; Stangor, 2014; Neufeld *et al.*, 2022). The social significance of food for this age group has been documented in previous research which suggests that adolescents use food as a medium through which to develop meaningful relationships with their peers (Neely *et al.*, 2014). However, the value placed on social relationships may increase the appeal of energy-dense foods which are readily available, and socially acceptable (Galván, 2013; Neufeld *et al.*, 2022). Research conducted to investigate the meaning and values associated with school packed lunches highlighted how young people choose well-known and fashionable brands to fit in with their peers and to avoid ridicule (Stead *et al.*, 2011). Further to this, many healthy food choices were considered by young people as clashing with adolescent values around socializing and enjoyment (Stead *et al.*, 2011; Strömmer *et al.*, 2021). Other qualitative research focussing on adolescents’ food choices outside of the home and school context has mainly focussed on specific outlet types including fast-food and takeaway outlets. Findings from two research studies conducted in London, UK suggest that fast-food and takeaway outlets are often viewed by young people as part of everyday life, valuable community assets and essential to the ability to live well (Thompson *et al.*, 2018; Burningham and Venn, 2022). Furthermore, consuming food from fast-food and takeaway outlets was described as providing a way to interact with their local environment as these outlets are affordable, accessible and abundant as well as being seen as a safe space to interact with their peers (Burningham and Venn, 2022).

Further research is needed to understand how social factors interact with adolescents’ use of wider aspects of the food environment outside of home and school and their collective impact on the foods adolescents buy and eat.

This research was designed to answer two research questions:

1. How do adolescents describe interacting with their social and food environments when purchasing their food?
2. How does the interplay between the social and physical food environment influence the healthfulness of food purchases made by adolescents?

## METHODS

### Study design

This study used an exploratory qualitative design to gain an understanding of how adolescents perceived, experienced and made sense of their food purchasing behaviours, and the environments in which these behaviours took place. Qualitative research methods were suitable for this study as they can gain a detailed understanding of individuals’ lived experiences and capture the complexities of everyday life (Braun and Clarke, 2013). This study is underpinned by a relativist ontology and subjective epistemology which propose that a single true reality exists but as knowledge is socially constructed and always interpreted through a frame of reference based on personal experience and insight, it can only ever be partially accessed (Punch, 2013; Dieronitou, 2014). This study is reported in line with the Journal Article Reporting Standards for Qualitative Research (Levitt *et al.*, 2018).

### Recruitment

A convenience sample of participants was recruited using a snowballing technique. Adolescents aged 11–18 years and attending secondary school or college in England were eligible to take part. Study details were circulated through stakeholders with connections to groups of adolescents e.g. local youth club leaders and teachers, and on social media. If an adolescent, and their friends, were interested in participating, they were asked to create a group and complete an online expression of interest form. The research team then contacted the potential participants with further details. The young people who took part in the study were unknown to the research team prior to conducting the interviews. After the initial recruitment phase, purposive sampling techniques (via local councils and charities) were used to target adolescents with a lower SES who had not yet been represented in the study.

Informed consent was received from all adolescents, or their parents, using an electronic form prior to participating in the research. Adolescents aged 16 years and older were able to consent themselves. For those aged under 16 years, parental consent was sought, and these adolescents provided informed assent.

### Data collection

Data were collected between October 2020 and April 2021. Prior to the qualitative data collection, participants completed a demographics form indicating their age, gender, ethnicity and SES. Home postcodes were collected to determine residential neighbourhood deprivation level. Questions from the Family Affluence Score (Currie *et al.*, 2008) were included to provide details about household-level SES.

Focus groups were the preferred qualitative data collection format because they allow participants to explore and clarify their views in the presence of others whose views may be similar or different (Kitzinger, 1994, 1995). The size of the focus groups varied between two and six participants and consisted of friends. Participants in each focus group had shared experiences of buying and eating food together. One individual interview was conducted to include a participant who did not wish to take part in a focus group. The make-up of each focus group is shown in Table 1.

**Table 1:** Participant characteristics for each focus group

	Number of participants	N boys/girls	Age, range	Home IMD, range	FAS, range
Group 1	3	3/0	15	10^a^	7–11
Group 2	4	3/1	15	9–10	10–11
Group 3	3	0/3	15	6–10	8–12
Group 4	5	0/5	17–18	5–10	12
Group 5	4	0/4	12–13	10	10–12
Group 6	2	0/2	14	10^a^	8–12
Group 7	4	2/2	17–18	6–8^a^	11
Group 8	4	1/3	17–18	9–10^a^	11–12
Interview 9	1	0/1	16	3	7
Group 10	4	0/4	15–16	4–8	10–11
Group 11	4	0/4	17	7–10	7–10
Group 12	4	4/0	17–18	2–8	10–11
Group 13	3	3/0	15–16	8–9^a^	6–12
Totals	45	16/29	12–18	2–10	6–12

FAS, Family Affluence Score.

^a^IMD score missing for 1 participant in each of these groups.

A semi-structured discussion guide (Supplementary Materials) was developed to ensure the discussions covered the topics of interest while allowing flexibility to follow up on topics that arose through the group conversation (DeJonckheere and Vaughn, 2019). The discussion guide comprised ‘open discovery’ questions which began with ‘what’ or ‘how’ to encourage participants to reflect on the topic and provide detailed responses from their personal perspective (Lawrence *et al.*, 2016). The discussion guide asked how participants perceived the food environments around their homes and schools, their use of different types of food outlets and their experiences in those outlets. The participants were not provided with any definitions of ‘healthy’ or ‘unhealthy’ foods but were free to explore their own perceptions of healthfulness. Where possible participants were asked to describe the types of foods they were discussing. The facilitator also used pictures of different types of food outlets (a fast-food outlet, convenience store and restaurant) to prompt discussion. Using pictures as part of semi-structured qualitative interviews is thought to sharpen participants’ memories of particular experiences and encourage more detailed responses about those experiences (Epstein *et al.*, 2006).

All focus groups, and the individual interview, were conducted online using Microsoft Teams, audio-recorded, and lasted between 47 and 62 min. Focus groups were facilitated by the lead author, who is a woman completing a PhD in public health nutrition with previous experience of conducting online focus groups with adolescents. A second researcher acted as an observer in the group interviews. After each focus group, the lead facilitator and observer discussed and made notes capturing their initial impressions of topics discussed.

### Data analysis

Audio-recordings were transcribed verbatim by an external transcription company. Participants did not comment on their final interview transcript. NVivo 12 (QSR International) software was used to organize and code the data. Inductive thematic analysis was conducted, following established guidelines (Braun and Clarke, 2006, 2013). The transcripts were read thoroughly by the lead author who made reflective notes on first impressions and meaning of the data. All transcripts were then coded inductively by the lead author with a new code being created for each new topic that arose during the coding. Quotes that fitted under more than one code were coded in all appropriate places. The coding was reviewed by the second author and detailed discussions were held between the two researchers to organize the codes into appropriate themes and subthemes. After this stage, other members of the research team were involved to reflect on the underlying meaning of the themes and the links between the themes to create a thematic map.

## RESULTS

### Participants characteristics

A total of 45 adolescents participated in 12 focus groups and one individual interview. Of these, 64% were girls and 80% were White British. Participant ages ranged from 12 to 18 years and most (62%) lived in areas categorized as the three least deprived deciles for neighbourhood disadvantage. The median Family Affluence Score was 11 (IQR 11,12; range 6 and 12) suggesting moderate to high levels of household-level SES. Participant characteristics are presented in Table 1, further details are found in Supplementary Material. While participants from across England were eligible to participate, most participants were based in the south of England as this is where the research team has strong connections with stakeholders such as charities, schools and youth clubs.

### Key findings

Five themes were identified from the data. The final coding framework can be found in Supplementary Material). Each theme is described below with illustrative participant quotes. The quotes have been edited slightly to remove superfluous material without altering the meaning.

#### Spending time with our friends is more important than the food

Social occasions surrounding adolescent food choices were described as more important than the food itself, however, food outlets played an important function by providing an enjoyable space for these social interactions to occur. Participants desired food outlets that were welcoming to people their age, comfortable and offered somewhere they could enjoy each other’s company. Large chain fast-food outlets were mostly named by all groups as suitable locations for these social interactions.

Participant (P): I try to go there, but in the end just kind of trail back to McDonald’s (laugh).

Facilitator (F): So, you kind of go to McDonald’s more often than other places?

P: Definitely.

P: For me, it’s quick food and it’s good when you’re with like a big group of people because there are loads of tables and stuff. Yeah. Just sit and talk with your friends really.—Group 5

Young people did not want to miss out on spending time with their friends if they did not have enough money to purchase food. It was common for young people to go to food outlets with friends but not purchase anything or only buy small inexpensive items, such as a drink. While this behaviour would not be appropriate in all food outlets, it was acceptable in fast-food restaurants because of the relaxed atmosphere. Participants enjoyed sharing food with each other as it was a way to involve friends who did not have money to buy food.

Fast-food outlets were often described as somewhere to get food but not a meal, while sit-down chain restaurants, such as Wagamama, or Nando’s, were seen as suitable locations for ‘proper meals’. Most eating occasions with friends were spontaneous, while a ‘proper meal’ was considered a special occasion and involved more planning, time, money and behaving more formally than they normally would with friends. Planning special occasions involving food was described more often by girls and by those from more affluent backgrounds.

P: There’s a really big difference between a fast-food restaurant and a sit-down restaurant, so I guess the sit-down one is the one where I would say, ‘This is my proper full meal. I’m having a whole pizza or a whole pasta thing,’ rather than one little burger… where that’s just, you’ve got it in your cardboard box and you can go.—Group 10

#### We spend our money on the things that are worth it

All participants, even those from more affluent backgrounds and those earning their own money, described having limited money to spend on food. Young people often thought healthy food was too expensive for them to purchase. Restaurants and cafes were seen to serve healthier food but were considered too expensive to visit regularly; fruit and vegetables were also viewed as expensive. Many participants were reluctant to spend their money on healthier foods because they were perceived as less enjoyable.

P: I also think unhealthy food’s always cheaper. So, like, Pret-A-Manger [chain sandwich outlet] is quite expensive for what you get, obviously, it is healthier, but when you’re like our age… you just go cheaper.—Group 5

Many participants described making use of deals and promotions to get the most for their money. Discounted, small-scale deals, often found on confectionary and snack foods, and meal deals (sandwich, snack and drink together for a discounted price) were considered to be particularly good for young people. Some participants also described making use of larger promotions when with their friends so they could share the food.

P: There’s these small-scale deals that I think are very good for like our age, at the one-two-three-pound mark. You wouldn’t really get, like, a ten-pound deal.

P: If you’re with friends you’re going to get like a ten-pound deal, but then there’s like obviously more in there. People can pay you back or whatever.—Group 2

Participants chose to visit food outlets they knew served food which fitted their limited budgets; having money-off vouchers and knowing which outlets provided ‘saver’ or ‘budget’ menu items was important when choosing an outlet.

P: When you get a bus ticket, they have the McDonald’s vouchers on the back. I guess a lot of people use them because it gets 99p off or something.

P: Adding on to that point, because we get the bus quite a lot, we get a lot of those McDonald’s vouchers. So, it’s just making us more want to go to unhealthy places because then we’re getting it even cheaper than it already is.—Group 5

Young people believed that unhealthy food was more likely to be included in promotions than healthier options, contributing to the view that healthy food was more expensive and out of their budget. Even though most participants said promotions made food more appealing, some expressed reluctance to buy healthy items even if they were on promotion.

P: Definitely on vegetables, fruits, you very rarely see them [promotions], unless they’re going out of date, but on crisps, like sugary drinks, pizza, things like that…you’re much more likely to get deals.—Group 4

Participants recognized that promotions often made them spend more money than intended but noted their view of ‘value’ included foods they liked and that were filling even if these were more expensive. They were also aware that they spent more money on food when out with their friends.

F: How much money would you normally spend when you’re by yourself or with friends?

P: Definitely more when with friends… I just feel when I’m on my own, I don’t feel like I need it. But there’s just something about being with friends, you want to treat yourself.—Group 2

#### We want our food choices to require little effort

Participants thought unhealthy foods were more readily available in the food outlets they visited, making not choosing them difficult. Participants thought some healthy choices were available in most outlets, but they tended not to choose these options.

P: I’ve never seen someone buy a salad in McDonald’s, to be honest.

P: Yeah, I think if you’re going to McDonald’s you’re not going there to be healthy.—Group 4

Young people wanted the act of purchasing food to be quick. They described rarely going to large supermarkets or sit-down restaurants because these required more time. Young people also wanted food that was ready for immediate consumption and single-serve portions were appealing so food did not have to be carried around. Many young people described buying items placed in prominent store locations and saw this as beneficial as it meant they did not have to spend time looking around the store. Participants also thought food stores made it easy for them to find promotions and deals through eye-catching signage and prominent.

P: Well at the front in Londis [chain of convenience stores in the UK], there’s always offers for four packs of Mars bars and that, which are a quid [£1]. And that kind of just reminds you that you’re going in there to buy… they’re kind of wanting you to buy something unhealthy.—Group 13

Some young people discussed how colour and wording used on packaging made some food items appear healthier than they really were. They noted that nutrition information, such as calorie labelling, was difficult to interpret. Some participants thought if companies are willing to provide health claims on packaging, the item must be healthy.

P: Things that on the front of them say, ‘reduced sugar’ or ‘reduced calories’ or ‘healthier option.’ You know, making it really obvious that this is supposedly a ‘health’ option, which they often aren’t. If it’s making that quite a priority on the packaging. I’m probably more likely to be enticed to that.—Group 10

Some participants felt that more could be done by the government and food companies to encourage healthier food choices and thought there was a responsibility for them to do so. Without being prompted about government policy, participants described being aware of restrictions on the sales of energy drinks but were critical that these policies were not implemented in all store types.

P: If it’s a convenience store, you can buy energy drinks and all that. You can just get a lot of stuff that you wouldn’t get in like Tesco’s.

F: What kind of different drinks do you mean? Do they not sell energy drinks in Tesco’s?

P: They have an age limit on them.

P: I think there’s no point in there being the [energy drink restrictions] ‘cos if I wanted an energy drink I’d just go to like Premier [chain convenience store] or something. Obviously, I can’t get it in Tesco’s [large supermarket chain], but I can still buy it—Group 1

#### We choose the foods we know

All participants visited familiar and popular chain food outlets or convenience shops located near their homes and/or schools. Participants preferred places they knew and were reluctant to try new outlets. Young people spoke about having a ‘usual order’ in certain food outlets which was known to their friends; they liked outlets that made few changes to their menu.

P: Also with McDonald’s, it’s like you always know what you want because they never change their menu really and it’s always good, consistent food.—Group 5

Advertising and branding built a sense of familiarity. Young people described frequently seeing advertising for food outlets in places like bus stops, bus tickets, social media, TV and YouTube videos.

P: There was the whole [influencer] burger thing which McDonald’s did to, I guess, appeal to younger people [group laughter]

P: So this rapper and he’s quite popular, so they worked with McDonald’s and made this burger that he usually orders

P: There was no difference in the burger. It was something like a regular but just without lettuce or something.

P: But it’s got his name on it… everyone just—everyone would just buy it.—Group 12

It was common for young people and their peers, to post images of foods to social media. Even though this was not a formal type of advertising, young people described it as increasing the desirability of certain foods.

P: Say someone ordered a Domino’s, and they post it on their Instagram story, and it makes you think, ‘oh, I want a Domino’s now.’ It reminds you of the taste of the Domino’s.—Group 13

Young people discussed brands that were easy to identify by their colours and packaging branding and stated how seeing branding made them want to buy certain food even though they didn’t necessarily think of it as formal advertising.

P: I don’t really think, to be honest, that they need to advertise it that much, because it’s so popular. Also, I think the main advertising that makes me want to get it is just seeing people with their McDonald’s bags. It just makes me think, ‘Oh, that looks so good. I want it,’ you know? I don’t really pay attention to the bus things like [name] does.—Group 5

#### Freedom of making our own food decisions

A theme that appeared to underlie all the other themes was young people's use of food to express their independence from their parents and other adults. Young people described having few opportunities to make their own decisions.

P: We only have a little bit of freedom so we just use it on food we like…Maybe when we have more responsibility when we’re older to buy, to have choices more often, then we’ll think about being healthy more.—Group 3

Even though participants described that they wanted to be healthy, they wanted to use their growing independence to buy foods they really liked even if these decisions did not meet the approval of their parents. Many participants expressed reluctance to buy food they had at home, and this reason was often provided for not buying foods they considered to be healthy for themselves.

P: When you’re outside with your friends and you go to a shop like Tesco’s [supermarket chain], you don’t like really think, ‘Oh, I’m going to buy an apple,’ ‘cause you normally have like apples and bananas and oranges and stuff like that at home.—Group 1

Older participants described freedom in their ability to drive and earn money. They had much greater independence in choosing what to eat, where to go and which friends to take on social outings. Younger participants described having fewer occasions to socialize with their friends with food but those they did have felt more special.

P: I think it’s just the freedom of driving. Like I’m often giving somebody a lift, and then it’ll just come up, do you fancy a Maccies [McDonalds] trip—Group 7

P: For our birthday, we were allowed to go into town or something when we were year five or six and that was like ‘Wow!’ Like, ‘We’re so independent now.’ But now it’s like something that you don’t even, like, second guess, it’s just normal.—Group 5

### Thematic map

**Fig. 1:**
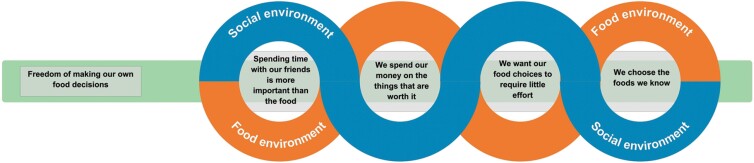
Thematic map reflecting the combined influence of factors from the social and physical food environments on adolescents’ independent food choices.

Figure 1 is a thematic diagram showing how the five themes inter-relate. Social factors, represented by the yellow line, were often at the forefront of what adolescents valued when purchasing food but factors from the food environment, represented by the blue line, shaped and often improved the quality of their social interactions. All four of these themes formed part of how the young people made their choices when purchasing food. Food needed to be social, present financial and social value, be familiar to them and their peers, and require minimal effort and quick decisions. These themes are not distinct from one another but instead are intertwined and play a role in shaping each other. Underlying these four main themes is the cross-cutting theme relating to independence. Food was a means by which young people wanted to express their autonomy and independence. Adolescents described experiencing increased independence from the home and their parents, and this played a role in how, when and where they spent time with their friends and created opportunities to make decisions about food.

## DISCUSSION

### Summary of findings and interpretations

This study engaged adolescents aged between 12 and 18 years in discussions about their experiences when making independent food choices. The findings demonstrate the value placed on social interactions with peers and the important role this plays in determining which food outlets adolescents decide to visit and which foods they buy. Young people seek out environments that will enhance their social interactions with peers. Some food outlets, mainly fast-food outlets, are uniquely suited to providing a welcoming, social space with affordable, familiar and popular food. The thread of independence was an important underlying factor in all the themes identified. Young people wanted to feel independent from adults, and purchasing their own food provided an opportunity to do this. Adolescents used the freedom they had to make their own food choices as a time to ‘rebel’ from the rules their parents had about food. Foods that are heavily marketed, cheap and socially accepted by peers were often those that parents did not approve of, making them more appealing to young people.

In line with previous research, this study showed that adolescents seek out spaces to socialize on their own terms and in their own time around food (Wills *et al.*, 2016; Kelly *et al.*, 2021; Murray and Wills, 2021). Particular food outlets offer desirable spaces that allow young people to enact their collective independence which is one reason why out-of-home and out-of-school environments are so appealing to this age group (Wills *et al.*, 2016). Global chain outlets, fast-food outlets in particular, were recognized in this study as offering a comfortable place for this age group to socialize. This finding is problematic for public health because adolescents are drawn to socializing in outlets known for serving energy-dense, nutrient-poor foods. Previous research investigating the use of corporate chain cafes by people of various ethnicities suggested that global chain food outlets are attractive to a wider range of individuals, including younger people, as the etiquette of how to use this space and behave is widely known and easy to understand (Jones *et al.*, 2015). The current study adds to this literature by describing how adolescents regularly visit the same global chain food outlets, as well as regularly ordering the same food items; this fulfils their desire for simple, straightforward and quick food choices, but avoids the need for spontaneous decisions which have the potential to cause embarrassment in front of their peers and other patrons of the food outlet. These findings suggest that alternative, more health-promoting environments that provide the familiarity and comfort of global chain brands are required to allow adolescents to socialize with their peers and express their independence in a space that does not compromise their health.

Adolescents’ desire to buy cheap and affordable food has been well documented in the literature (Caraher *et al.*, 2016; Kelly *et al.*, 2021; Ziegler *et al.*, 2021). Whilst participants in this study expressed their desire to get a good deal, they also explained they valued more than just price when making these purchases. Adolescents’ willingness to spend more when with friends, and for the foods they enjoyed most, demonstrated the social value adolescents placed on food. Isaacs *et al.* describe how low-income parents purchase foods available in their food environments to fulfil a wide range of needs such as social experiences, enjoyment and treats, and to keep with cultural traditions; their low incomes meant other ways to meet these needs were unaffordable. Like low-income parents, adolescents may be limited to food outlets which predominantly serve unhealthy foods because these are the outlets recognized as providing a good social experience and providing foods that are recognizable while also fitting with their limited budgets. The findings from this study support previous calls for food environment interventions and policies to be re-designed not only to support nutritionally better food choices but to also support other aspects of human–well-being such as social and emotional needs (Hawkes *et al.*, 2020; Isaacs *et al.*, 2022).

This study adds to the current literature by providing insights into how adolescents interact with the environments inside food outlets to make their food choices. The young people in this study reported using smaller food stores, such as convenience stores, more frequently because they were easier and quicker to navigate. Some young people discussed how the foods they often wanted to buy were placed in prominent, easy-to-find locations such as the front of the store. In addition, young people described making use of promotions, sometimes sharing them with friends, to get better value for money. Young people described using vouchers for fast-food outlets that were found on the back of bus tickets. These types of vouchers particularly target young people and those from more disadvantaged backgrounds as they tend to rely more on public transport. Previous research with retailers has documented how some adapt their practices in line with their adolescent customers’ desires. Such adaptations included implementing simple ordering systems, observing adolescent purchases and stocking similar items to encourage alternative purchases, and ensuring that hot or fresh foods were ready at specific times of day, such as after school, when adolescents are most likely to be visiting the outlet, encouraging quick food purchases (Wills *et al.*, 2016). Young people may be particularly sensitive to environments which promote unhealthy foods because the brain’s reward system develops earlier than the region responsible of behavioural control meaning they may find it difficult to resist environmental cues for cheap, heavily promoted unhealthy foods and instead make reasoned decisions focussed on benefitting their health (Konrad *et al.*, 2013).

This study and prior evidence illustrate how food is a means through which adolescents can develop autonomy, social connections with others and independence; skills that are important for their emotional and psychological development (Dahl *et al*., 2018). However, the food environments in which young people may be developing these important skills often bombard them with prompts that encourage the consumption of unhealthy foods. More needs to be done to support adolescents to develop these skills in environments that are supportive of their health and well-being.

### Implications for policy

The results of this qualitative study provide important insights into the role government food environment policies may have on the food choices of adolescents. Below, the findings have been interpreted in relation to policies introduced as part of the UK Government’s Childhood Obesity Strategy. Evidence from this study suggests that in order for these policies to be effective in young people, refinements may be needed to capture the complexities of adolescents’ food choices. Three examples are provided below:

1. Mandatory policies are required

Adolescents in this study expressed expectations for the government to help people their age stay healthy and demonstrated an awareness of existing strategies that are intended to encourage healthier dietary behaviours. Participants knew some retailers would not sell energy drinks to people under 16 years but were under the impression that this was implemented by the government rather than a voluntary industry strategy. Many young people viewed this strategy as pointless because they knew the outlets that would and would not sell energy drinks to people their age. This example demonstrates the need for government policy to be effectively implemented and enforced across different types of food outlets. As previous research has shown energy drink consumption is increasing among adolescents from deprived backgrounds (Vogel *et al.*, 2022), mandatory restrictions on the sale of energy drinks have the potential to help address dietary inequalities.

2) Policies are required in the food outlets adolescents use most frequently and on items they regularly purchase

The findings from this study suggest that UK Government regulations to restrict the prominent placement and multibuy promotions could have a beneficial impact on adolescents’ food choices. However, these regulations exclude small retailers with under 50 employees or stores that are smaller than 185.8 m^2^ (Department of Health and Social Care, 2021b). These small out-of-scope stores can continue to market unhealthy foods and it is therefore possible that adolescents will not receive the full benefit offered by these policies. This gap in the policy coverage may potentially widen inequalities as populations known to have poorer diets (i.e. young people, older adults and socioeconomically disadvantaged families) often rely on convenience stores to purchase food (Muir *et al.*, 2023). In addition, currently no placement or promotion strategies are being implemented in fast-food outlets, another outlet type frequently used by this age group.

Furthermore, the participants in this study described meal deals (sandwich, snack and drink promotions) as a commonly purchased promotion among people their age because they provide ready-to-eat food in a small-scale, affordable promotion. The promotions regulation, due to be implemented in October 2025, excludes these types of deals because they are categorized as ‘lunch options for adults to consume on the go’ (Department of Health and Social Care, 2021b). Finding ways to include healthier products that are still appealing to young people in these types of small-scale deal may be one way to improve the healthfulness of adolescents’ food choices.

1. Introduce policies to combat the persuasive influence of branding

This study suggests that the UK Government restrictions on advertising, now scheduled for introduction in January 2025 (Department for Digital, Culture, Media and Sport and Department of Health and Social Care, 2021), are important because many participants reported seeing adverts for unhealthy foods in online content such as videos and social media feeds that directly targeted their age group. Similar to the promotion restrictions, these advertising restrictions will be based on HFSS foods defined using the UK's Nutrient Profile Model (Department for Health, 2011). However, no limits will be placed on the advertising of food branding provided no specific HFSS products are identifiable in the advert; for example, the globally recognized KFC brand can be included in an advert as long as no HFSS products are shown (Department for Digital, Culture, Media and Sport and Department of Health and Social Care, 2021). The findings from this study suggest this loophole may be a downfall in the effectiveness of the advertising restrictions for young people. Participants described how seeing high-profile food branding made them think of, and want to buy, foods which they considered to be unhealthy. Hence, tougher restrictions on the advertising of brands that sell predominately HFSS foods should be considered in future regulation refinements rather than only applying to individual food items. The issue of brand advertising has been highlighted as an area of concern in the recent WHO guidelines to protect children from harmful food marketing and was marked as an area for future consideration (World Health Organization, 2023). In addition, restricting advertising on HFSS foods and brands presents a potential double benefit for health by providing advertising spaces for more health-promoting foods which may help to build levels of familiarity and social acceptability towards these foods. Young people in this study described how it was common for people their age to post images of food on social media and how this increased their desire to buy and eat similar foods, in the same way as formal advertising. Such peer-to-peer promotion may be another way that young people use food to socialize, but it is difficult for government regulation to address this type of promotion. However, introducing restrictions on formal advertising for HFSS foods and brands may start to shift social norms and the acceptability of those foods and, in turn, start to alter the types of food young people use to socialize.

The examples above highlight the need for thorough evaluation of how food environment policies impact the food choices of adolescents. To ensure policies are as effective as possible at improving the diet and health of younger populations, adolescents should be included in policy-making decisions and evaluation plans.

### Strengths and limitations

This qualitative study has provided insight into the experiences of adolescents who are starting to make more of their own food choices. The focus groups were conducted with adolescents of mainly White British ethnicity residing in the south of England and most were from more advantaged socio-economic backgrounds. Although these factors may limit the generalizability of the findings, it is likely that the implications for government policy are also relevant to young people from lower socio-economic backgrounds. The food environment policies being implemented in the UK are considered low-agency interventions, and therefore thought to be more equitable due to reduced requirements for cognitive and psychological abilities as well as time and material engagement from the end user (Adams *et al.*, 2016). It has been argued that such policies are therefore particularly important in shaping food choices for those from more disadvantaged communities who will have limited resources to seek out healthier choices (Adams *et al.*, 2016; Theis and White, 2021).

The study was conducted between October 2020 and April 2021. Due to the COVID-19 pandemic, it is likely that adolescents may have been using food outlets less frequently or in different ways during these times. COVID-19 was discussed during the focus groups but did not alter the overall themes identified in the study.

## CONCLUSIONS

This study reveals that social environments and food environments are highly interrelated in their influence on adolescents’ independent food choices. The findings highlight that new food environment policies need to include evaluations that specifically consider their impact on the food purchases and diets of adolescents. Efforts to improve food environments, and shape social norms and attitudes around food choices, may be most effective if they aim to harness widely shared adolescent values beyond those relating to nutrition or health. Adolescents should be active partners in shaping local and national policies which alter food environments to ensure such policies meet their needs.

## Supplementary Material

daad097_suppl_Supplementary_Material
