# Supplementary material for: The interplay between social and food environments on UK adolescents’ food choices: implications for policy
Source: Health Promot Int. 2023 Aug 30;38(4):daad097. doi: 10.1093/heapro/daad097 (PMC10468012; doi:10.1093/heapro/daad097)

**Supplementary Material: Summary of participant characteristics**

| **Characteristics** | **Boys (n=16)** | **Girls (n=29)** |
| --- | --- | --- |
| **Age, n (%)**  12-13  14-16  17-18 | 0 (0) 9 (56) 7 (44) | 4 (14) 11 (38) 14 (48) |
| **Ethnicity, n (%)**  White British  Indian  Filipino  White other | 12 (75) 3 (19) 1 (6) 0 (0) | 24 (83) 3 (10) 1 (3) 1 (3) |
| **Home neighbourhood deprivation (IMD deciles), n (%)**  1-4 (most disadvantaged)  5-7  8-10 (most advantaged)  Missing | 1 (6) 2 (13)  10 (63)  3 (19) | 2 (7) 7 (24) 18 (62) 2 (7) |
| **Family Affluence Score***  Median (IQR) | 10.50 (10,11) | 11 (11,12) |
| *Family affluence score does not include the question about foreign family holidays as the ability for such trips was limited due to the COVID pandemic. | | |

**Supplementary Material: Coding frame showing themes and subthemes**

| 1. **Spending time with our friends is more important than the food** |
| --- |
| Choosing a space to all be together |
| Eating is often secondary to the social activity |
| Going for a meal is a more formal occasion |
| Social status associated with some food outlets |
| 1. **We spend our money on the things that are worth it** |
| Choosing food outlets that are cheap |
| Cheap food is often less healthy |
| Being careful about how they spend their own money |
| Using deals and promotions to get more for the money |
| Willing to spend extra for a better experience |
| 1. **We want our food choices to require little effort** |
| Healthy foods are less accessible |
| Unhealthy food is more available in the food outlets visited by young people |
| Choosing unhealthy foods that are in prominent locations |
| Young people want quick and hassle-free food |
| Foods can appear healthier than they really are |
| More could be done to make healthy eating easier |
| 1. **We choose the foods we know** |
| Going to well-known chain food outlets |
| Buying the same food every time they visit a particular outlet |
| Influence of advertising on choosing food |
| Influence of social media and celebrity endorsement |
| Buying recognisable brands |
| 1. **Freedom of making our own food decisions** |
| Desire to buy food that is not available at home |
| Value given to being healthy |
| Capitalising on freedom to make own food choices by choosing treat items |
| Increased access to more food outlets with increasing impendence |

**Supplementary Material: Discussion Guide**

Question Route:

**Your local area**

- What food outlets are close to your home/school?
- How healthy are the food outlets that are close by?
- How easy is it to visit food outlets in your local area without an adult to take you there?

**Food outlets you visit**

- What food outlets do you visit most often?
- What days do you typically visit *[FOOD OUTLET]*?
- What time of day do you typically visit *[FOOD OUTLET]*?
- What makes you choose to go to this food outlet?
- How many people your age regularly visit *[FOOD OUTLET]*?
- What are the things you like/dislike about this food outlet?

**Food purchases**

- How much money do you typically spend each time you visit food outlets without your parents/guardian?
- How do you get the money you spend? Is it pocket money, savings, given by parents etc?
- What sort of foods do you typically buy for yourself?

[USE PHOTO PROMPTS- offers, advertising, special promotions, packaging, food placement, price, vouchers.]

- How did you decide what you are going to purchase?
- When do you normally eat the things you buy? Breakfast/lunch/dinner/ a snack?

**Other people**

- Who are you typically with when you visit your favourite food outlet?
- What foods do they typically buy?
- How often you tend to buy different things when you are with different people?

**Photo prompts:**


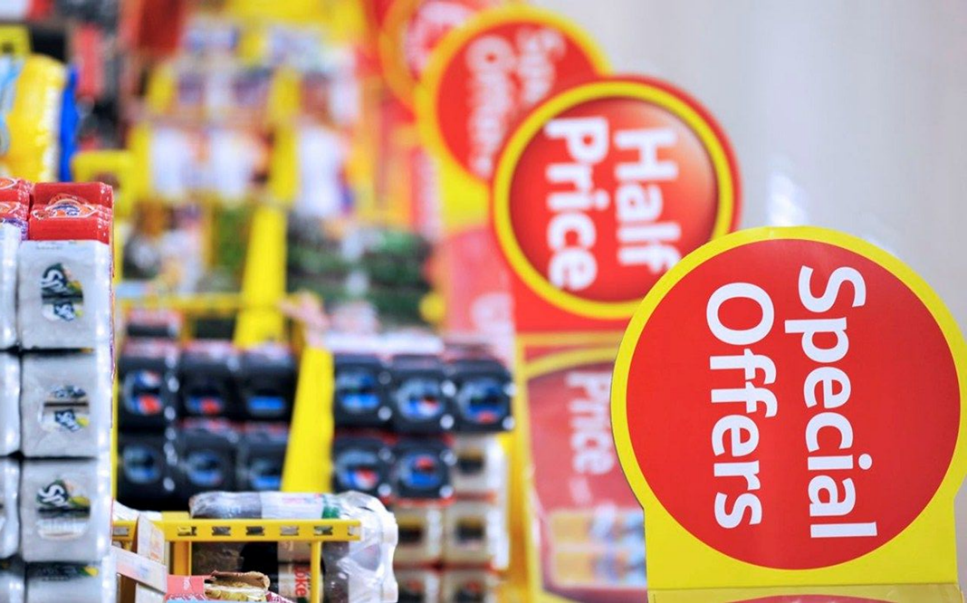


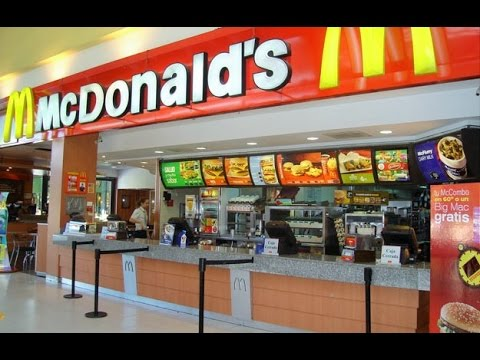


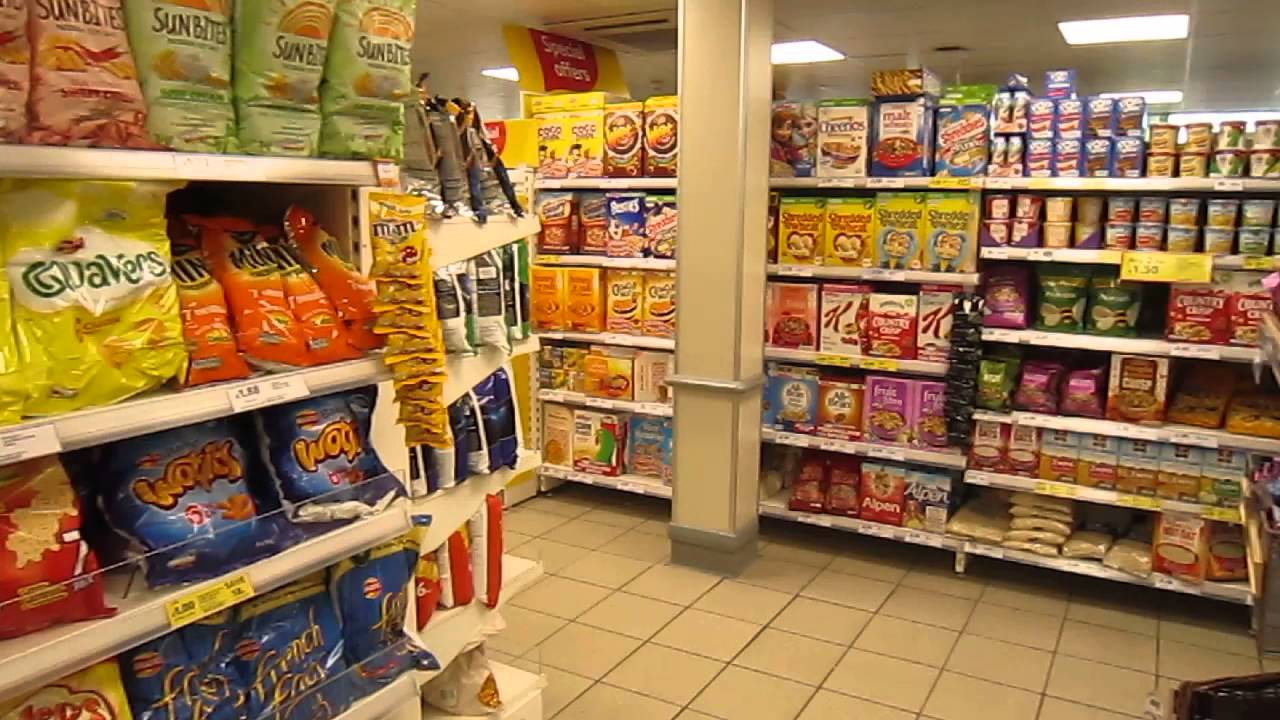


#
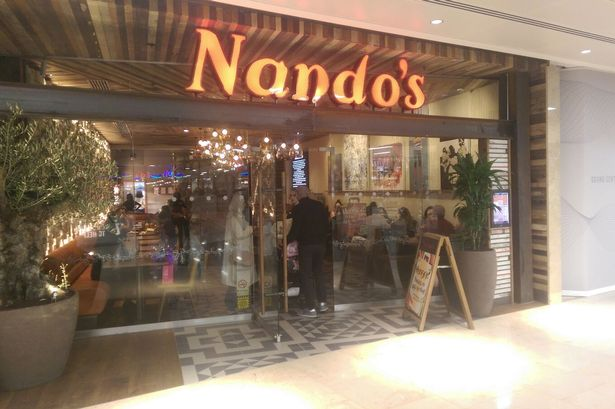

Supplement: daad097_suppl_Supplementary_Material [file daad097_suppl_supplementary_material.docx]
